# Successful conservative management of hepatic portal venous gas complicating severe immune-related gastrointestinal injury during pembrolizumab-based concurrent chemoradiotherapy for cervical cancer: a mechanistic case report

**DOI:** 10.3389/fonc.2026.1942174

**Published:** 2026-09-30

**Authors:** Miwa Minamitani, Hideaki Tsuyoshi, Tatsushi Naito, Yasunari Nakamoto, Makoto Orisaka

**Affiliations:** 1 Department of Obstetrics and Gynecology, Faculty of Medical Sciences, University of Fukui, Fukui, Japan; 2 Second Department of Internal Medicine, Faculty of Medical Sciences, University of Fukui, Fukui, Japan

**Keywords:** cervical cancer, concurrent chemoradiotherapy, gastritis, hepatic portal venous gas, immune-related adverse events, pembrolizumab

## Abstract

Concurrent chemoradiotherapy (CCRT) combined with pembrolizumab has become the standard treatment for high-risk locally advanced cervical cancer, increasing the need for prompt recognition of immune-related adverse events (irAEs). We report a rare case of severe gastrointestinal irAE complicated by hepatic portal venous gas (HPVG) during pembrolizumab-based CCRT. A 72-year-old woman with FIGO 2018 stage IIIC1r cervical squamous cell carcinoma developed persistent diarrhea and anorexia during treatment, followed by sudden hematemesis after the first brachytherapy session. Upper gastrointestinal endoscopy revealed diffuse gastric mucosal necrosis, while contrast-enhanced computed tomography demonstrated HPVG, intramural gastric gas, and diffuse gastrointestinal wall edema without evidence of intestinal ischemia. Gastric biopsy showed marked intraepithelial lymphocytic infiltration predominantly composed of CD8-positive T cells, consistent with pembrolizumab-induced gastrointestinal irAE. High-dose corticosteroid therapy and supportive conservative management resulted in rapid clinical and radiological resolution, avoiding surgical intervention. This case highlights that gastrointestinal irAEs may mimic conventional CCRT-related toxicities, delaying diagnosis. Furthermore, HPVG should not be considered synonymous with intestinal necrosis. Careful integration of clinical, radiological, endoscopic, and pathological findings is essential to distinguish immune-mediated gastrointestinal injury from ischemic disease and to guide appropriate management.

## 1 Introduction

Concurrent chemoradiotherapy (CCRT) combined with the anti-programmed cell death protein 1 (PD-1) antibody pembrolizumab has recently become the new standard of care for patients with high-risk locally advanced cervical cancer following the KEYNOTE-A18 trial, which demonstrated significant improvements in both progression-free and overall survival (1). As the use of immune checkpoint inhibitors (ICIs) continues to expand, the early recognition and appropriate management of immune-related adverse events (irAEs) have become increasingly important clinical challenges (2, 3).

Gastrointestinal irAEs are among the most common toxicities associated with ICI therapy, with colitis representing the predominant manifestation (4). In contrast, gastric involvement is uncommon. Moreover, gastrointestinal symptoms such as anorexia, nausea, and diarrhea overlap substantially with the adverse effects of chemotherapy and radiotherapy, making the timely diagnosis of irAEs particularly challenging during CCRT. Although severe cases may result in extensive mucosal injury or necrotic changes, the concurrent presence of hepatic portal venous gas (HPVG) is exceedingly rare (5).

HPVG has traditionally been regarded as a radiological finding associated with intestinal ischemia and necrosis. Historically, HPVG has been considered a potentially life-threatening finding, particularly in the setting of bowel ischemia or necrosis, and has often prompted urgent surgical consideration. However, recent evidence suggests that HPVG itself is not invariably associated with irreversible intestinal damage, and its clinical significance is largely determined by the underlying etiology. Accumulating evidence indicates that it may also occur in a wide range of conditions, including inflammatory disorders and iatrogenic causes (5, 6).

Here, we report a case of severe gastrointestinal irAE that developed during pembrolizumab-based CCRT and was complicated by intramural gastric gas and HPVG. Histopathological examination of gastric biopsy specimens demonstrated predominant infiltration of CD8-positive T cells. The patient recovered with conservative management centered on high-dose corticosteroid therapy. This case provides important insights into the pathophysiology and clinical management of severe ICI-related gastrointestinal toxicity complicated by HPVG.

## 2 Case description

A 72-year-old postmenopausal woman (gravida 2, para 2; menopause at 45 years of age) with a history of hypertension and dyslipidemia presented with abnormal genital bleeding. After being diagnosed with cervical cancer at a referring hospital, she was transferred to our institution for further management. She had not undergone cervical cancer screening in recent years.

On admission, her height was 147 cm, weight 56 kg, and Eastern Cooperative Oncology Group (ECOG) performance status 0. Pelvic examination revealed a friable, highly vascular tumor replacing the cervix, while rectal examination demonstrated parametrial invasion extending to the pelvic wall. Transvaginal ultrasonography showed a 45-mm cervical mass without endometrial or adnexal abnormalities. Histopathological examination confirmed human papillomavirus (HPV)-associated squamous cell carcinoma. Serum carcinoembryonic antigen (CEA) was 1.6 ng/mL (reference value <5.0 ng/mL), whereas squamous cell carcinoma (SCC) antigen was elevated at 3.8 ng/mL (reference value <1.5 ng/mL). Chest radiography and electrocardiography were unremarkable.

Contrast-enhanced pelvic magnetic resonance imaging (MRI) demonstrated a 60-mm cervical tumor that was hypointense on both T1- and T2-weighted images with strong early contrast enhancement. The tumor extended to the lower third of the vagina and broadly contacted the bladder without evidence of mucosal invasion^18^.F-fluorodeoxyglucose positron emission tomography (FDG-PET)/MRI demonstrated FDG uptake in the primary lesion and bilateral iliac lymph nodes without distant metastases. The patient was diagnosed with the International Federation of Obstetrics and Gynecology (FIGO) 2018 stage IIIC1r cervical cancer, and pembrolizumab-based CCRT was initiated.

External beam radiotherapy was delivered using volumetric-modulated arc therapy (VMAT) with a TrueBeam linear accelerator (Varian Medical Systems). The irradiation targets included the uterus, parametrial tissues, ovarian regions, metastatic lymph nodes, and the pelvic lymphatic regions, including the common, internal, and external iliac, obturator, and presacral lymph node regions. The prescribed dose was 1.8 Gy per fraction for 28 fractions, for a total dose of 50.4 Gy. The stomach and upper gastrointestinal tract were outside the radiation field and were not included in the irradiated treatment volume. Weekly cisplatin (40 mg/m²) and pembrolizumab (150 mg every 3 weeks) were administered concurrently. Brachytherapy using a remote afterloading system (RALS) was planned at 12.0 Gy in two fractions; however, because severe gastrointestinal irAE developed after the first session, only one fraction of 6.0 Gy was administered, and subsequent vaginal brachytherapy was discontinued. Common Terminology Criteria for Adverse Events (CTCAE) grade 1 diarrhea (1–3 episodes/day) developed during the first treatment week but remained stable throughout treatment. After the fourth treatment cycle, the patient developed grade 4 neutropenia, grade 3 thrombocytopenia, pyelonephritis, and grade 3 anorexia, necessitating discontinuation of cisplatin. Contrast-enhanced computed tomography (CT) performed in the seventh week after treatment initiation showed no structural abnormalities explaining the persistent diarrhea or anorexia. Enteral nutrition with a semi-digested formula was therefore initiated. One week after the first RALS session, the patient developed sudden hematemesis in the ninth treatment week (**Figure 1**).

**Figure 1:**
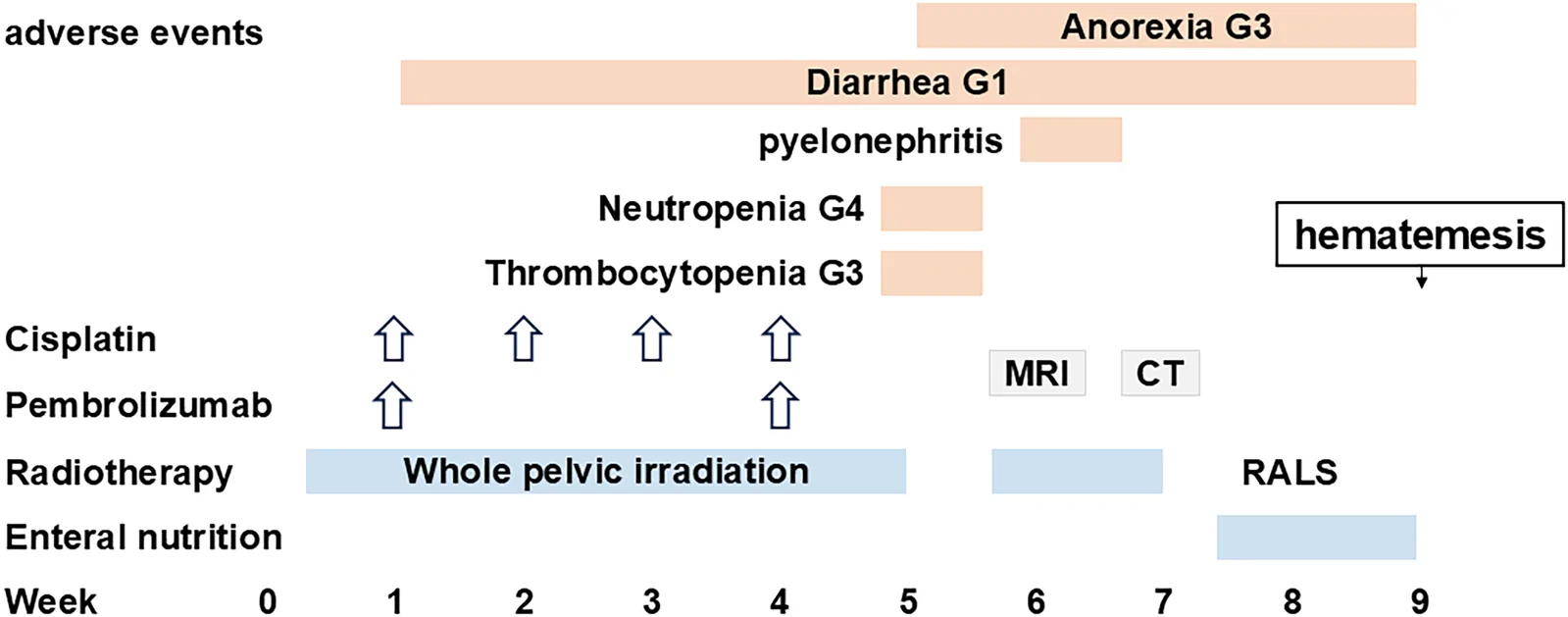
Clinical course before the onset of severe gastrointestinal immune-related adverse event. MRI, magnetic resonance imaging; CT, computed tomography; RALS, remote afterloading system; G, Grade.

On presentation, her blood pressure was 114/76 mmHg, pulse rate 98 beats/min, and body temperature 36.2 °C. The abdomen was soft, flat, and non-tender. Laboratory testing revealed a white blood cell count (WBC) of 27,200/μL, C-reactive protein (CRP) level of 16.4 mg/dL, and lactate dehydrogenase (LDH) level of 258 U/L (reference range <222 U/L). Despite the marked inflammatory response, she was afebrile, hemodynamically stable, and in good general condition, with no signs of systemic infection or peritoneal irritation. Blood cultures, stool cultures, testing for Clostridioides difficile, and serum procalcitonin (PCT) were not performed before initiation of corticosteroid therapy and broad-spectrum antibiotics, given the absence of clinical findings suggestive of systemic infection. Emergency upper gastrointestinal endoscopy demonstrated diffuse dark-red gastric mucosa with extensive mucosal necrosis and sloughing (**Figure 2A**). Contrast-enhanced CT revealed HPVG, marked gastric wall edema with intramural gas, and diffuse edematous thickening of both the small and large intestines (**Figures 2B, C**). No vascular occlusion, bowel wall hypoenhancement, thromboembolism, mechanical obstruction, or other findings suggestive of intestinal ischemia were identified. The patient remained afebrile and hemodynamically stable, and the abdomen was soft and non-tender without peritoneal signs. Although the WBC count and CRP level were markedly elevated, LDH was only mildly elevated (258 U/L). Given the recent administration of pembrolizumab, characteristic endoscopic findings, and the absence of radiologic or clinical evidence of intestinal ischemia, CTCAE grade 4 gastrointestinal irAE complicated by HPVG was considered the most likely diagnosis. After multidisciplinary consultation with the gastrointestinal surgery and gastroenterology teams, conservative management was selected because there were no clinical or radiologic evidence suggesting transmural ischemia or irreversible bowel necrosis, and surgical intervention was considered unlikely to provide benefit in the setting of extensive gastrointestinal involvement.

**Figure 2:**
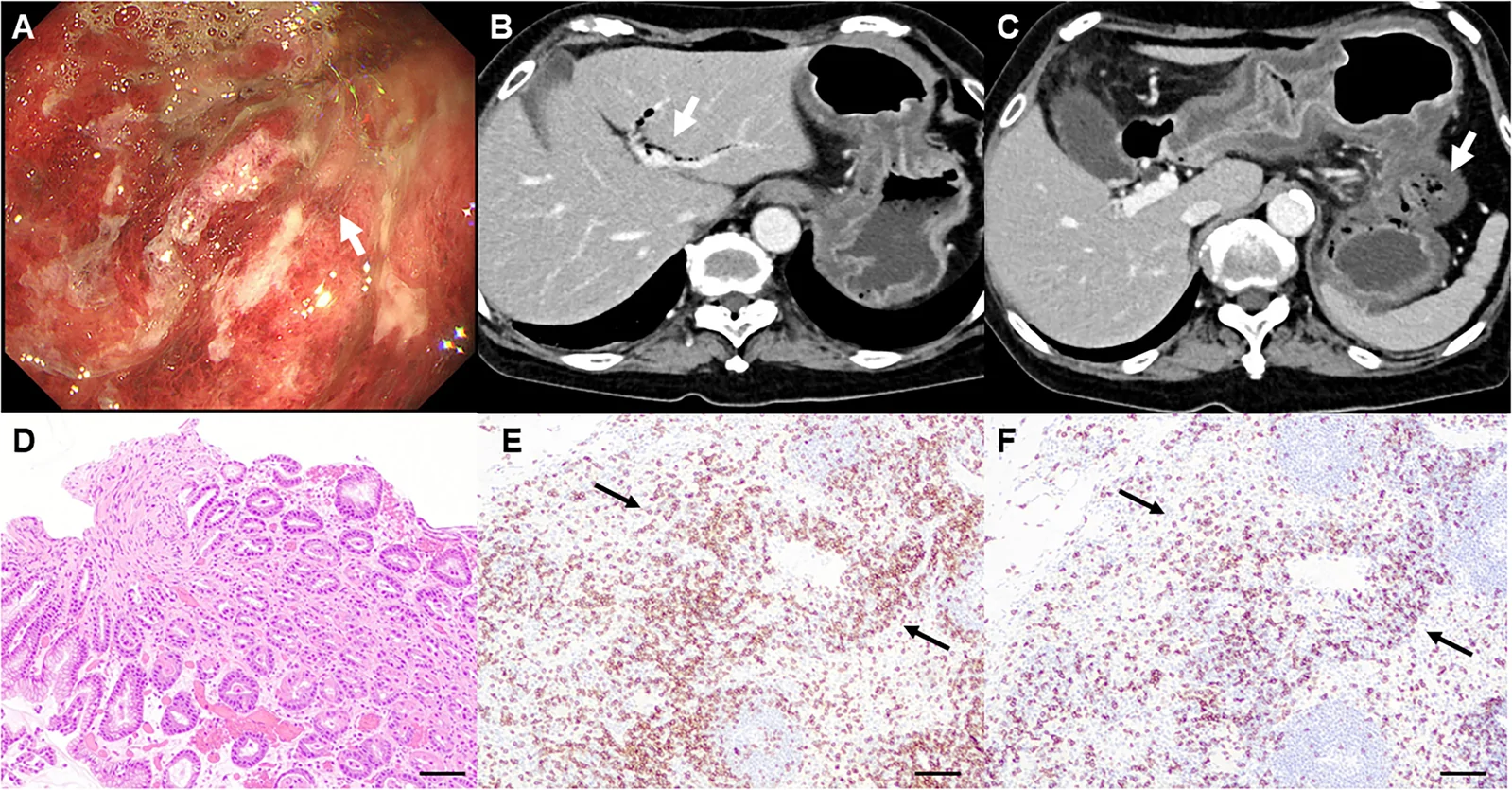
Endoscopic, computed tomography, and histopathological findings of severe gastrointestinal irAE. **(A)** Upper gastrointestinal endoscopy showing diffuse dark-red discoloration of the gastric mucosa with extensive mucosal necrosis and sloughing (arrow). **(B)** Contrast-enhanced computed tomography demonstrating hepatic portal venous gas (arrow). **(C)** Contrast-enhanced computed tomography showing marked edematous thickening of the gastric wall with intramural gastric gas (arrow) and diffuse bowel wall edema without evidence of intestinal ischemia or vascular occlusion. **(D)** Hematoxylin and eosin staining of the gastric mucosa showing marked intraepithelial lymphocytic infiltration within the glandular epithelium (magnification ×100). **(E)** Immunohistochemical staining demonstrating infiltration of CD3-positive T lymphocytes (magnification ×100). **(F)** Immunohistochemical staining showing that the infiltrating T lymphocytes were predominantly CD8-positive (magnification ×100). Arrows in **(E)** and **(F)** indicate CD3-positive and CD8-positive T lymphocytes, respectively. Scale bars indicate 100 μm in **(D–F)**.

Treatment consisted of bowel rest, total parenteral nutrition, cefmetazole sodium, proton pump inhibitors (intravenous omeprazole followed by oral esomeprazole magnesium), and high-dose intravenous prednisolone (100 mg/day, approximately 1.8 mg/kg/day based on a body weight of 56 kg). The inflammatory response improved rapidly after treatment initiation, and hematemesis did not recur. Three weeks later, follow-up contrast-enhanced CT demonstrated complete resolution of HPVG and intramural gastric gas, with marked improvement in gastric and intestinal wall edema. Repeat upper gastrointestinal endoscopy showed complete resolution of the dark-red necrotic mucosa. Histopathological examination of gastric biopsy specimens demonstrated prominent intraepithelial lymphocytic infiltration on hematoxylin and eosin (HE) staining (**Figure 2D**). Neither prominent apoptotic epithelial injury nor crypt dropout was observed. Immunohistochemistry showed that most CD3-positive lymphocytes (**Figure 2E**) were CD8-positive (**Figure 2F**), supporting the diagnosis of pembrolizumab-associated gastrointestinal irAE.

After clinical symptoms and inflammatory findings improved, intravenous prednisolone was gradually tapered to 30 mg/day, followed by oral prednisolone, which was gradually tapered from 20 mg/day to 5 mg/day and discontinued seven weeks after initiation (**Figure 3**). During the fifth week of steroid therapy, the patient developed acute calculous cholecystitis. Abdominal ultrasonography and contrast-enhanced CT demonstrated gallstones with findings consistent with acute cholecystitis. Percutaneous transhepatic gallbladder drainage (PTGBD) and endoscopic retrograde gallbladder drainage (ERGBD) were performed, after which her clinical symptoms and inflammatory response improved. Pembrolizumab was permanently discontinued because of the grade 4 irAE, and rechallenge was not attempted. Follow-up contrast-enhanced CT performed three weeks after steroid discontinuation demonstrated no recurrence of HPVG, intramural gastric gas, or intestinal edema. No relapse of gastrointestinal irAE has been observed during six months of follow-up.

**Figure 3:**
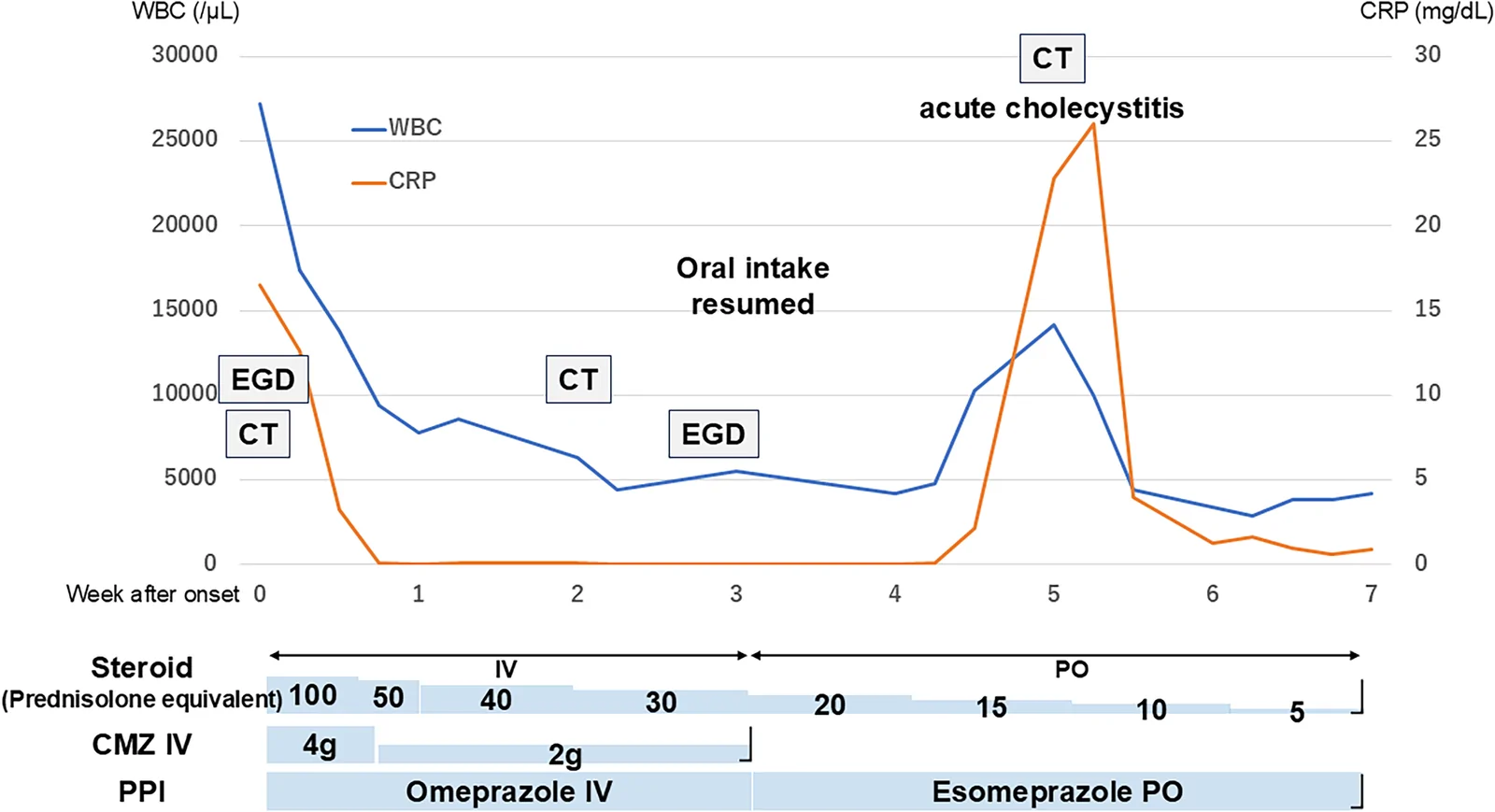
Clinical course after the onset of severe gastrointestinal immune-related adverse event. MRI, magnetic resonance imaging; CT, computed tomography; EGD, esophagogastroduodenoscopy; WBC, white blood cell; CRP, C-reactive protein; IV, intravenous administration; PO, oral administration; CMZ, cefmetazole sodium; PPI, proton pump inhibitor.

## 3 Discussion

This case describes a severe pembrolizumab-related gastrointestinal irAE complicated by intramural gastric gas and HPVG during CCRT. Although colitis is the most common gastrointestinal toxicity associated with ICIs, severe gastric involvement is uncommon. The unique features of this case are the coexistence of extensive gastric mucosal injury and HPVG, together with pathological findings supporting an immune-mediated mechanism.

The diagnosis of gastrointestinal irAE was supported by the clinical course, endoscopic findings, histopathology, and response to treatment. ICI-related gastritis demonstrates diverse endoscopic features, including erythema, edema, erosions, ulceration, and necrosis (4), while histopathological examination typically reveals intraepithelial lymphocytic infiltration, predominantly by CD8-positive T cells (4, 7). In the present case, gastric biopsy showed marked intraepithelial lymphocytic infiltration with a predominance of CD8-positive T cells. Neither prominent apoptotic epithelial injury nor conspicuous crypt dropout was observed. The rapid resolution of inflammation, endoscopic abnormalities, and HPVG following high-dose corticosteroid therapy further supports an immune-mediated pathogenesis. Although pelvic radiotherapy was administered concurrently, the stomach and upper gastrointestinal tract were outside the radiation field and were not included in the irradiated treatment volume. Therefore, the predominant gastric involvement, the absence of therapeutic irradiation to the stomach, the characteristic pathological findings, and the rapid response to corticosteroid therapy strongly favored pembrolizumab-associated gastrointestinal irAE over radiation-induced injury. Furthermore, the gastric lesion was anatomically remote from the vaginal brachytherapy field, making a direct radiation-induced gastric injury unlikely.

The corticosteroid dose and tapering strategy in this case in accordance with the ASCO guideline recommendations for severe ICI-related gastrointestinal toxicity (2). The patient’s body weight was 56 kg, and high-dose intravenous prednisolone was initiated at 100 mg/day, corresponding to approximately 1.8 mg/kg/day. Given the extensive gastric mucosal necrosis, HPVG, marked inflammatory response, and CTCAE grade 4 gastrointestinal toxicity, this dose was considered appropriate for severe immune-mediated gastrointestinal injury. After clinical symptoms and inflammatory findings improved, intravenous prednisolone was gradually tapered to 30 mg/day, followed by oral prednisolone at 20–5 mg/day, and was discontinued seven weeks after initiation. The ASCO guideline recommends corticosteroid tapering over at least 4–6 weeks after improvement to grade 1 or lower 2. Thus, the seven-week tapering period in this case was consistent with the recommended duration and was selected cautiously to minimize the risk of recurrence given the severity and extensive gastric mucosal involvement.

Several alternative diagnoses were considered in the differential diagnosis of the severe gastric and gastrointestinal injury observed in this case. In particular, infectious causes could not be completely excluded before high-dose corticosteroid therapy. Blood and stool cultures, testing for Clostridioides difficile, cytomegalovirus, Helicobacter pylori, and procalcitonin were not obtained before treatment. Although the patient was hemodynamically stable and afebrile, these findings alone cannot exclude infection, particularly in patients receiving anticancer therapy. Current ASCO and ESMO guidelines recommend prompt evaluation for infectious etiologies in suspected severe immune-related gastrointestinal toxicity before or concurrently with immunosuppressive treatment when clinically feasible (2, 3). In this case, the severity of the gastric mucosal injury and HPVG prompted immediate high-dose corticosteroid therapy based on the overall clinical, endoscopic, and radiological findings. Nevertheless, the lack of pretreatment pathogen screening represents an important limitation and underscores the importance of rapid infectious evaluation, even in clinically stable patients, before initiating high-dose immunosuppression.

Stress-related mucosal injury was also considered, but the extensive gastric mucosal necrosis, prominent CD8-positive lymphocytic infiltration, and rapid response to corticosteroid therapy favored an immune-mediated process. Chemotherapy-associated mucosal injury was considered because of prior cisplatin administration; however, cisplatin had been discontinued approximately five weeks before the onset of hematemesis, making direct cisplatin-induced gastric injury less likely. Radiation-induced gastric injury, including a potential scatter effect, was considered unlikely because the stomach and upper gastrointestinal tract were outside the external beam radiation field and were not included in the irradiated treatment volume. Furthermore, the stomach was anatomically remote from the vaginal brachytherapy field. Neutropenic enterocolitis was also considered given the history of grade 4 neutropenia; however, the patient was no longer neutropenic at the time of HPVG onset, and the predominant lesion was gastric rather than ileocecal. Finally, intestinal ischemia was carefully evaluated because of the presence of HPVG. Contrast-enhanced CT showed no vascular occlusion, bowel wall hypoenhancement, thromboembolism, mechanical obstruction, or other evidence of intestinal ischemia, and the patient remained hemodynamically stable without peritoneal signs. Taken together, these clinical, temporal, imaging, endoscopic, and histopathological findings made infectious, stress-related, chemotherapy-associated, radiation-induced, neutropenic, and ischemic injuries less likely and supported pembrolizumab-associated gastrointestinal irAE as the most likely explanation for the gastric and gastrointestinal injury.

An important clinical lesson from this case is that gastrointestinal irAEs may initially mimic the expected toxicities of CCRT. The patient developed grade 1 diarrhea soon after treatment initiation, followed by grade 3 anorexia. Because these symptoms are common during chemoradiotherapy and no structural abnormalities were identified on contrast-enhanced CT in the seventh treatment week, they were initially managed as treatment-related adverse effects. In retrospect, however, they may have represented prodromal manifestations of severe gastrointestinal irAE. Persistent or worsening gastrointestinal symptoms during ICI-containing treatment should therefore prompt consideration of irAE, with early endoscopic evaluation when clinically indicated (2, 3).

The presence of HPVG posed an important diagnostic challenge because it is traditionally associated with intestinal ischemia and necrosis. However, HPVG is now recognized to occur in a variety of inflammatory, infectious, and iatrogenic conditions, and its clinical significance depends on the underlying cause (5). Decisions regarding surgical intervention should therefore be based on the overall clinical picture rather than imaging findings alone. In the present case, contrast-enhanced CT demonstrated no vascular occlusion, bowel wall hypoenhancement, thromboembolism, mechanical obstruction, or other evidence of intestinal ischemia. The patient remained hemodynamically stable, with a soft and non-tender abdomen and no peritoneal signs. Taken together, the absence of radiologic evidence of impaired intestinal perfusion, together with the stable clinical condition and absence of peritoneal irritation, made transmural ischemia with irreversible bowel necrosis less likely. In addition, infection is an important differential diagnosis in patients with severe gastrointestinal inflammation and HPVG. Microbiological testing was not performed in this case. At the time of presentation, the patient was afebrile, hemodynamically stable, and in good general condition, without clinical evidence of systemic infection or peritoneal irritation. Although an infectious etiology could not be completely excluded because blood cultures, stool cultures, Clostridioides difficile testing, PCT measurement, and specific testing for cytomegalovirus and Helicobacter pylori were not performed, the clinical course, characteristic endoscopic findings, histopathological evidence of predominant CD8-positive lymphocytic infiltration, and rapid response to corticosteroid therapy collectively supported an immune-mediated mechanism as the most likely explanation for the gastrointestinal injury. Consequently, conservative management centered on immunosuppressive therapy was selected, resulting in complete clinical recovery without surgery.

Enteral nutrition may also have contributed to the development of HPVG. Approximately two weeks before its onset, the patient had started a semi-digested enteral formula. Enteral nutrition has been reported as a potential precipitating factor for pneumatosis intestinalis (PI) and HPVG through increased intraluminal pressure and mucosal injury, facilitating the migration of luminal gas into the bowel wall and portal venous system (8). PI is characterized by gas within the bowel wall, with gas cysts typically located in the submucosa or subserosa when intestinal necrosis is absent (9). Although the clinicopathological findings strongly suggest that severe immune-mediated mucosal injury was the primary mechanism underlying HPVG in this case, enteral nutrition may have further promoted gas migration through the already damaged mucosa. Thus, the development of HPVG was likely multifactorial (10).

Only a few cases of ICI-related gastrointestinal toxicity complicated by HPVG have been reported. One previous report described pneumatosis intestinalis and HPVG during pembrolizumab-containing chemotherapy for advanced esophageal cancer. After ICI rechallenge, the patient died, and autopsy demonstrated intestinal injury with predominant CD8-positive lymphocyte infiltration (6). Compared with that case, our patient had predominantly gastric rather than small intestinal involvement, underwent clinicopathological evaluation during the acute phase by gastric biopsy rather than postmortem examination, and recovered with corticosteroid-based conservative management without surgical intervention. This case adds to the limited literature on ICI-related gastrointestinal injury complicated by HPVG, particularly cases with predominant gastric involvement and pathological confirmation during the active phase (**Table 1**).

**Table 1:** Comparison of reported ICI-related gastrointestinal adverse events complicated by HPVG.

Study	Cancer type	ICI	Combination therapy	Time to onset	Symptom	General condition at onset	Predominant gastrointestinal lesion	Treatment	Steroid response	Outcome	Histopathology
Tashiro, et al., 2025 (6)	Esophageal cancer	Pembrolizumab	5-FU+CDDP	12w	abdominal pain and distension	lost consciousness and developed shock	small intestine	antibiotics and steroids	No response	Died the following day	Autopsy showing CD8-positive lymphocytic infiltration in the small intestine
Present case	Cervical cancer	Pembrolizumab	CCRT (CDDP)	9w	Hematemesis	stable	Stomach, large and small intestine	antibiotics and steroids	Clinical improvement	Recovered	Gastric biopsy showing predominant CD8-positive T-cell infiltration

HPVG, hepatic portal venous gas; ICI, Immune-checkpoint inhibitor; CT, Computed Tomography; 5-FU, 5-fluorouracil; CDDP, Cisplatin; w, week; CCRT, concurrent chemoradiotherapy.

## 4 Conclusion

Persistent or progressive gastrointestinal symptoms during ICI-containing CCRT should prompt consideration of gastrointestinal irAE in addition to conventional treatment-related toxicities. Likewise, HPVG should not be regarded as synonymous with intestinal necrosis. Careful integration of the clinical presentation, imaging, endoscopic findings, and pathology is essential for appropriate management. This case demonstrates that even severe gastrointestinal irAE complicated by HPVG can be successfully managed without surgery when ischemia is carefully excluded and immunosuppressive therapy is initiated promptly.

## 5 Patient perspective

The patient reported considerable anxiety when she developed sudden hematemesis during concurrent chemoradiotherapy and was informed that hepatic portal venous gas (HPVG) had been detected on computed tomography. She was concerned that the condition might require emergency surgery and questioned whether anticancer treatment could be continued. After receiving an explanation that the gastrointestinal injury was considered an immune-related adverse event associated with pembrolizumab and that conservative treatment with high-dose corticosteroids was appropriate, she agreed to the proposed management. As her symptoms, endoscopic findings, and imaging abnormalities gradually resolved without surgical intervention, she expressed relief that she had recovered from this potentially serious complication. She also stated that she better understood the importance of promptly reporting persistent gastrointestinal symptoms during immune checkpoint inhibitor therapy. Written informed consent was obtained from the patient for publication of this case report and any accompanying images.

## Data Availability

The original contributions presented in the study are included in the article/supplementary material, further inquiries can be directed to the corresponding author/s.
